# A Precautionary Tale: Individual Decision Making in the Time of COVID-19

**DOI:** 10.3390/ijerph20054597

**Published:** 2023-03-05

**Authors:** Ann Pearman, MacKenzie L. Hughes, Clara W. Coblenz, Emily L. Smith, Shevaun D. Neupert

**Affiliations:** 1 Department of Psychiatry, MetroHealth Medical Center, 2500 MetroHealth Drive, Cleveland, OH 44109, USA; 2 School of Psychology, Georgia Institute of Technology, Atlanta, GA 30332, USA; 3 Department of Psychology, North Carolina State University, Raleigh, NC 27695-7650, USA

**Keywords:** decision making, perceived risk, health, daily diary, COVID-19

## Abstract

Precaution taking is an important part of managing COVID-19 and has been since the start of the pandemic. Guided by the Health Belief Model, two studies conducted during the beginning of the COVID-19 pandemic aimed to identify possible individual difference predictors of precautionary actions. Study 1 was an online, cross-sectional study using 763 adults aged 20–79 years old. Study 2, a 30-day daily diary study, examined daily precautions in 261 persons over the age of 55 years old. Study 1 and Study 2 indicated that COVID-19 knowledge predicted precautionary behaviors. Multilevel models from Study 2 indicated that daily increases in in-person interactions and leaving home were associated with decreases in precautions, but increases in disruption to routine were associated with increases in precautions. In both studies, including concurrent and lagged models in Study 2, significant interactions between information seeking and perceived risk suggested higher information seeking was related to higher precautions for those who consider themselves low risk. Findings highlight the burden of daily precautions and potentially modifiable factors of engagement in precautions.

## 1. Introduction

The outbreak of coronavirus disease 2019 (COVID-19) has put individuals around the world at risk of becoming sick and, in many cases, dying. The virus is highly transmissible between people, including people who are asymptomatic [1]. The highly contagious nature of COVID-19 has led to an exponential increase in cases and deaths around the world. At the time of data collection for the current study (April 2020–June 2020), the United States had 2,431,816 confirmed cases of COVID-19 and 123,979 COVID-19 related deaths [2]. The differences between cases and deaths in the United States compared to neighboring countries were profound. For instance, at the time of data collection for the current study, Canada had 102,231 confirmed cases with 8451 deaths [2]. Understanding American persons’ experiences and decision making during this time is an important step in improving the ongoing pandemic experience in the United States.

Early in the pandemic (late March–early April, 2020), communities and governments around the United States issued stay-at-home orders and most businesses, schools, and other public meeting areas closed while only essential businesses remained open to the public. Precautionary behaviors to prevent the spread of illness, such as wearing masks in public, were encouraged by the Center for Disease Control (CDC) and World Health Organization (WHO) but not were not mandated or enforced in the United States. It is now understood that slowing the spread of COVID-19 depends on public adherence to avoidant and preventative health behaviors [3]. Even though information related to how the disease is transmitted and how to prevent further spread of COVID-19 is widely available, not all individuals engage in precautionary behaviors. Identifying factors that may influence decision making related to the adoption of precautionary behaviors during the COVID-19 pandemic could provide insights into how to increase engagement in these behaviors. Using the Health Belief Model (HBM) [4] to guide our conceptualization, we examine endorsed precautionary actions at the individual level in two studies during the initial height of the pandemic in the United States.

The HBM was developed to understand the adoption of illness prevention behaviors that have potential to protect personal and community health. The model suggests that the likelihood of adopting precautionary health behaviors to prevent contracting and spreading a disease depends on several interrelated factors including perceived susceptibility of contracting the disease, perceived severity of the disease, and whether there are effective actions that can be taken to reduce susceptibility or severity if one becomes ill [4]. Perceived susceptibility and severity can indirectly be influenced by demographic and individual differences variables, such as socioeconomic status, social group-related norms, and knowledge of the disease [4]. For instance, it may be the case that people with more knowledge about COVID-19 perceive themselves as more susceptible to the disease [5] because of their enhanced understanding of COVID-19. The HBM also suggests that cues to action have a role in the adoption of precautionary behaviors. Cues to action, such as media and social media coverage as well as information presented by the government, can initiate individual-level health-promoting behavior such as engaging in recommended precautionary behaviors [6]. A person’s level of information seeking regarding a health threat, in this case COVID-19, may therefore also influence the action of engaging in precautionary behaviors.

Using the HBM framework as a guiding approach, we aimed to identify predictors of endorsement of COVID-19-related precautionary behaviors at the individual level. First, we examined the influence of age, other demographic characteristics (i.e., income and education), and knowledge about COVID-19 on precautionary behavior. We anticipated that more knowledge about COVID-19 would be related to greater endorsement of precautionary behaviors, as would higher income and education [7,8,9]. We included age in the analytic model due to probable age-related differences in the types of strategies people use to make health-related decisions. For instance, during the SARS outbreak, older adults and people with higher levels of education and income were most likely to take precautionary measures [8]. Age may also be positively related to precautionary actions because older adults generally have higher levels of perceived susceptibility to infection [10] particularly for COVID-19 infection [11]. The current two studies explore the relationship of age to precautionary behaviors both cross-sectionally (Study 1) and in concurrent and lagged daily diary analyses of only adults aged 55 and above (Study 2).

Based on the HBM, we also chose to examine the effects of individual beliefs, specifically perceived threat, by examining questions related to individuals’ perceived risk of avoiding or contracting COVID-19 on precautionary behavior. Perceived risk of COVID-19 is high around the world [12], and higher levels of perceived risk and perceived severity of COVID-19—also measured as perceived threat—tend to be related to the adoption of precautionary behaviors [7,13]. In addition, we investigated people’s beliefs in terms of skepticism and doubt about the veracity of the existence of a pandemic. We predicted that if people think the media reports about the pandemic are overblown or even untrue, they will engage in fewer precautionary behaviors [14].

In addition to perceived threat, we examined whether the extent to which pandemic-related stressor appraisals in the form of disruption to individuals’ daily routines is related to precautionary behaviors. Many people have had to adjust their schedules and daily routines to comply with recommended actions to prevent further spread of COVID-19 [11]. For example, more people are working from home, which may represent precautionary decision making by the individual, but it may also represent precautionary decisions by employers. We believed that understanding the relationship between disruptions to daily routine and endorsed precautions may provide insight into the pandemic burden [15].

Finally, to understand media-related cues to action, we examined how COVID-19-related information-seeking behavior influenced the adoption of precautionary behaviors at the individual level. Previous HBM research has demonstrated that risk perception and subsequent precautionary measures taken during illness outbreaks do not solely depend on the threat of the illness or the number of new cases but can also depend on information gathered from a variety of sources. For instance, information and misinformation shared through social media and other news outlets influence individuals’ perceptions of the seriousness of COVID-19 [16,17] and the value of recommended precautions [9]. During the influenza A (H1N1) outbreak, the perceived susceptibility of contracting the illness was positively related to people’s levels of media consumption covering the pandemic [18]. That is, the more people learned about the disease from media sources, the higher they perceived their own risk, which led to increased engagement in precaution taking. Reintjes et al. (2016) also showed that changes in public risk perception over time were more aligned with trends in pandemic-related media coverage than trends shown epidemiologically [19]. These findings highlight the need to better understand how information seeking can affect risk perception and decision making related to behavioral responses to a pandemic. This is especially true with the “fake news” messaging that has been prevalent in the media [20].

Examining determinants of precautionary behaviors during COVID-19 could help identify factors for intervention that influence the use of precautionary behavior [13]. In addition to helping avoid disease, precautionary behaviors also predict well-being [21]. The current work is focused on enhancing our understanding of factors influencing the endorsement of COVID-19-related precautionary behaviors using two studies. Study 1, a cross-sectional sample of adults ranging in age from 20 to 79 years old, seeks to identify the role of information seeking and personal perception of risk on protective behavior aimed at preventing the spread of COVID-19. Study 2, a daily diary study of adults aged 55 years and older, was designed to test the same predictors of behavior in Study 1 on a day-to-day basis specifically in a sample of middle-age and older adults and to also examine the influence of these predictors on precautionary behavior over time, including changes in precautions from one day to the next.

Based on the HBM and early studies on COVID-19, we hypothesized that endorsement of protective behaviors would be predicted by older age, high levels of education, income, information seeking, COVID-19 knowledge, perceived susceptibility of developing COVID-19, and low levels of beliefs in fake news, confidence in ability to avoid COVID-19, and number of in-person interactions. In Study 2, we examined how these processes unfold within a person over time as a way of exploring decision making about precautions on a day-to-day basis. For Study 2, we also predicted that leaving home on a given day would be directly related to reductions in precautionary behaviors and that increases in information seeking would be associated with concurrent day and subsequent day increases in precautionary behavior. In line with HBM, we also expected that the effects of information seeking would depend upon levels of perceived risk.

## 2. Materials and Methods

Two types of data were collected in these studies. Study 1 used a cross-sectional design to examine perceptions of risk and health-related behaviors from individuals across the adult lifespan. Study 2 used a daily diary approach to collect repeated assessments of similar COVID-19-related perceptions and behaviors from individuals aged 55 and over. This age group was chosen because of their higher risk profile in developing and dying from COVID-19 [22].

### 2.1. Participants

Both studies, Study 1 (cross-sectional) and Study 2 (daily diary), used Amazon Mechanical Turk (MTurk) for online recruitment and data were collected through the Qualtrics platform. To be eligible to participate, participants had to be living in the United States and be native English speakers. Participants had to be at least 18 years old to be eligible for Study 1 and at least 55 years old to be eligible for Study 2. Individuals were excluded from analyses if they were healthcare workers, diagnosed with dementia, or if they scored zero points on a COVID-19 knowledge quiz [11]. Participants provided electronic informed consent to indicate that they agreed to and understood the study procedures. The final sample in Study 1 included 763 people ages 20–79 (M = 38.74, SD = 11.51), 40.1% female, and from 48 U.S. states. The final sample in Study 2 included 261 people ages 55–79 (M = 64.29, SD = 5.20), 67.8% female, and from 41 states. See Table 1 for sample characteristics. Both studies were approved by the Georgia Institute of Technology Institutional Review Board.

### 2.2. Procedure

#### 2.2.1. Study 1

Participants completed the survey one time. Fifty Human Intelligence Tasks (HITS) were released on MTurk approximately every three days between 20 March and 14 May, 2020. Completing the cross-sectional survey took approximately 25 min, and participants were compensated $3.

#### 2.2.2. Study 2

Individuals ages 55 and over completed up to 30 consecutive days of diaries. Twelve staggered waves of MTurk HITS were released every several days between 1 April 2020 and 26 June 2020. Approximately 25 participants were in each wave. To be eligible for the study, participants had to complete the Day 1 diary and then agree to participate in the diary collection over the next 29 days. Completing the first diary took approximately 30 min, and the remaining diaries took approximately 15 min to complete. Participants were compensated $2 for completing the Day 1 diary and the Day 30 diary and compensated $1 for every daily diary they completed during the study. These are standard rates for this type of survey in the United States.

### 2.3. Measures

Measures were identical across the two studies, except the information-seeking items had different response scales between Study 1 and Study 2, and Study 1 did not have a question about leaving home that day. For information seeking, Study 1 used a scale ranging from 1 (never) to 5 (always), whereas Study 2 used a scale ranging from 1 (I did not use this source in the past 24 h) to 5 (7 or more times in the past 24 h).

#### 2.3.1. Precautionary Behaviors

As the outcome indicator of health action, a list of 14 potential precautionary behaviors was developed based on previous research on pandemics [10] and CDC recommendations on COVID-19 [23]. Example precautionary behaviors included wearing a mask, washing hands, and avoiding large gatherings of people (see Table 2 for all items). To measure endorsement of precautionary behaviors, participants responded (yes/no) to the items on the list that were relevant to them in general (Study 1) or in the last 24 h (Study 2). A mean score was created with higher scores indicating a greater use of precautionary behaviors. This score was then multiplied by 100 to create a percentage of precautionary behaviors endorsed.

#### 2.3.2. Demographics

Income, age, and highest educational degree were included because of their association with precautionary behavior [4,24].

#### 2.3.3. COVID-19 Knowledge and Beliefs Questionnaire [11]

To examine the role of existing COVID-19 knowledge and beliefs on precautionary behaviors, a 35-item questionnaire was used to identify both knowledge about COVID-19 and its symptoms as well as beliefs about the pandemic [11,25]. The knowledge portion of the questionnaire includes twenty-nine items based on information gathered from the CDC and WHO websites in March 2020. Participants responded (agree/disagree/don’t know) to questions related to the COVID-19 pandemic, including potential symptoms of coronavirus and recommendations to contain the spread of the illness. Due to the CDC’s addition of new symptoms during the data collection period, four knowledge questions were not included in the final score. Two additional items were excluded from the knowledge score due to their similarity with another item on the quiz, yielding 23 items. Correct responses received 1 point and incorrect or “don’t know” responses received 0 points. A sum score was created with higher scores reflecting greater levels of COVID-19 knowledge.

The beliefs portion of this questionnaire includes six items designed to identify skepticism about the COVID-19 pandemic (i.e., fake news beliefs). Example items include, “COVID-19 is not really different from the common cold”, “COVID-19 is being widely exaggerated by the press”, and “COVID-19 is pretty much ‘fake news’”. These questions used the same response scale described above (agree/disagree/don’t know). A sum score was created with higher scores reflecting higher levels of beliefs in the COVID-19 pandemic being fake news (Study 1, α = .78; Study 2, Day 1 α = .74).

#### 2.3.4. Perceived Susceptibility

Two questions examined the effect of perceived susceptibility to COVID-19. Participants answered the question “What do you think is the risk of you developing COVID-19?” on a 5-point scale ranging from 1 (very low) to 5 (very high). Participants also responded to a statement, “Rate your confidence level in your ability to avoid COVID-19” on a scale ranging from 1 (I feel I will not be able to avoid infection) to 5 (I feel confident I can avoid infection).

#### 2.3.5. Information Seeking

As a measure of action cues, we included a series of questions about the frequency in which participants used various sources to obtain pandemic-related information. The seven potential categories of information included websites, social media (e.g., Twitter), radio, TV, health officials (i.e., COVID-19 testing site), print materials (e.g., newspapers), and talking to others. For Study 1, participants answered the question, “How often have you used the following sources to get information about COVID-19?” on a 5-point scale ranging from 1 (never) to 5 (always). For Study 2, individuals were asked “How often in the past 24 h did you use the following sources to get information about COVID-19?” on a 5-point scale ranging from 1 (I did not use this source in the past 24 h) to 5 (7 or more times). A mean score was calculated with higher scores reflecting higher levels of COVID-19 information seeking.

#### 2.3.6. Daily Behaviors

To expand our understanding of the effects of daily behaviors and experiences as cues to action to precautionary behavior, we included three items regarding daily routine.

**Appraisal of Daily Routine Disruption.** Participants in both studies indicated the extent to which COVID-19 affected their daily routine in the past 24 h on a scale ranging from 1 (not at all) to 4 (a lot).

**In-Person Interactions.** Participants in both studies answered the question, “How many people have you interacted in-person within the past 24 h (including those you live with)?”

**Leaving Home.** In Study 2, participants also responded (0 = no, 1 = yes) to “Have you left your home in the past 24 h?”

### 2.4. Analysis

#### 2.4.1. Study 1

Correlations of the study variables are reported. In addition, a regression analysis was conducted using endorsed precautions as the outcome variable.

#### 2.4.2. Study 2

We analyzed the daily diary data using multilevel modeling (MLM) [26] because the data are nested (days nested within people) and we are interested in intraindividual (within-person) variability, that is, people’s fluctuations around their own mean. MLM was implemented using SAS Institute (1997) Proc Mixed with the REML estimation method. We used the equations below for concurrent models.

Level 1: Daily precautions*_it_* = β_0*it*_ + β_1*it*_ (info seeking) + β_2*it*_ (interactions) + β_3*it*_ (avoid confidence) + β_4*it*_ (appraisal) + β_5*it*_ (leave home) + r*_it_*

Level 2: β_0*i*_ = γ_00_ + γ_01_ (knowledge score) + γ_02_ (degree) + γ_03_ (age) + γ_04_ (income) + γ_05_ (avg. info seeking) + γ_06_ (fake news beliefs) + γ_07_ (COVID risk) + u_0*i*_

β_1i =_ γ_10_ + γ_11_ (COVID risk)

β_2i =_ γ_20_

β_3i =_ γ_30_

β_4i =_ γ_40_

β_5i =_ γ_50_

γ_10_–γ_50_ refer to the daily within-person associations between information seeking (info seeking), number of in-person interactions (interactions), self-rated confidence in ability to avoid COVID-19 (avoid confidence), appraisal of disruption to daily routine in the past 24 h (appraisal), whether or not one left home (leave home), and daily precautions. Daily information seeking was person-mean centered by adding each person’s mean score across study days as a covariate at Level 2 (person-level) to adjust for the fact that some individuals may seek more or less information on a regular basis than others. γ_00_ refers to the grand-mean score of precautions. γ_01_–γ_07_ represent the between-person main effects of COVID-19 knowledge score, educational degree, age, income, person-mean information seeking, fake news beliefs, and perceived COVID-19 risk, respectively. γ_11_ represents the cross-level interaction between daily information seeking and perceived risk, where the within-person relationship between daily information seeking and precautions is moderated by differences in perceived risk. The unexplained variance at the within- and between-person levels is modeled by r_it_ and u_0i_, respectively.

We also conducted lagged models by including in the same equations the precautions (the dependent variable) of the previous day as a predictor at Level 1, following procedures detailed in Grzywacz et al. (2004) and Neupert et al. (2006) [27,28]. The effect of central interest is still the cross-level interaction, but the interpretation of the within-person relationship shifted to represent the change in precautions from one day to the next.

## 3. Results

Regarding endorsed precautions in both studies, a large proportion of the samples indicated that they were engaging in preventative behaviors, such as avoiding small and large gatherings of people and washing hands (see Table 2 for the percentages of endorsed precautions). Table 3 shows study descriptive statistics.

### 3.1. Study 1

Table 4 (above the diagonal) shows the correlations among the Study 1 variables. The intercorrelations in Table 4 show there was a relatively weak overlap among the predictor variables in both studies, reducing concern of multicollinearity. Significant positive correlations with precautionary behaviors included income, knowledge, perceived risk of COVID-19, appraisal of daily routine disruption, and information-seeking behavior. Significant negative correlations with endorsed precautionary behaviors included the number of in-person interactions that day and fake news beliefs about COVID-19. Income and scores on the knowledge quiz were also negatively related to endorsed belief in fake news. 

**Figure 1:**
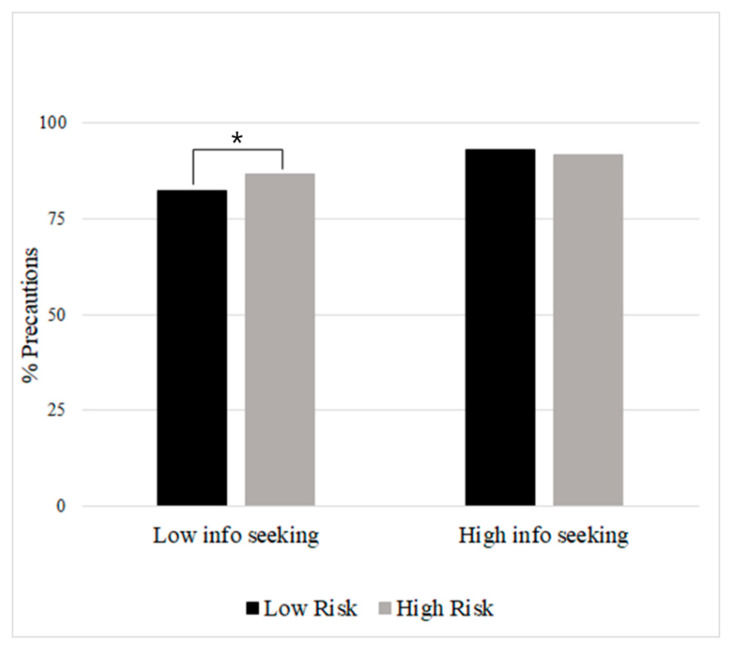
Information Seeking X Perceived COVID-19 Risk Interaction for Endorsed Precautionary Behaviors in Study 1. Note. All study covariates included in model. Low/high information seeking = M ± 1 SD. Perceived COVID-19 risk = M ± 1 SD. Persons who engaged in less information seeking and perceived themselves as at lower risk were the least likely to engage in precautionary behaviors. Asterisk (*) indicates *p* < .05 difference.

The regression analysis with precautionary behavior as the outcome variable is detailed in Table 5. Significant main effects included COVID-19 knowledge (higher knowledge, more precautions), appraisal of disruption to routine (higher disruption, more precautions), interactions (fewer in-person interactions that day, more precautions), and information seeking (more information seeking, more precautions). There was a significant interaction between perceived risk of developing COVID-19 and information seeking and on endorsed precautions. To better understand this interaction, we split both perceived risk and information seeking into two groups (high/low) using a median split (see Figure 1). High information seeking was associated with more endorsed precautions for those with both perceived risk groups. However, low information seeking in those with low perceived risk was associated with fewer precautions than those with higher perceived risk. This suggests that people who perceive themselves as lower risk and do not seek as much information are the least likely to engage in precautions. Study 2 examines these relationships within persons in concurrent day and lagged analyses.

### 3.2. Study 2

**Figure 2:**
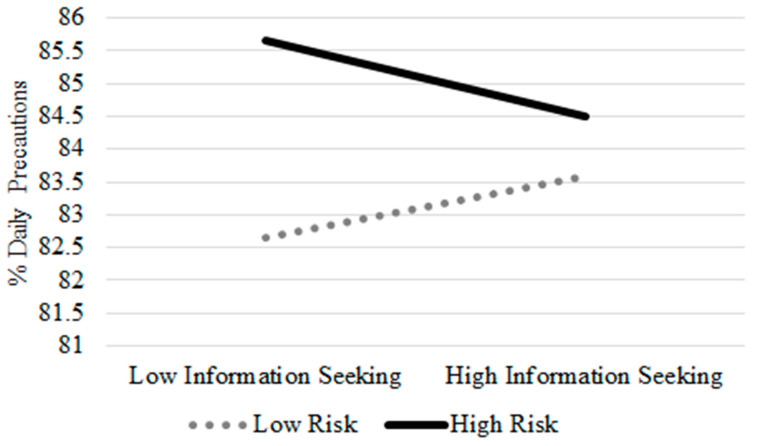
Cross-Level Interaction of Daily Information Seeking and Individual Differences in Perceived Risk of Developing COVID-19. Note. All study covariates included in model. Similar to Study 1, persons who were high in perceived risk but low in information seeking tended to endorse the most daily precautions particularly when compared to those low in perceived risk. The pattern above applies to the concurrent day model as well as the lagged model predicting changes in precautions from one day to the next.

Table 4 (below the diagonal) shows the results of the correlation analyses from the Day 1 diaries. Similar to Study 1, there was little concern of multicollinearity, as the intercorrelations show there is relatively weak overlap among the predictor variables. An unconditional model revealed that 85% of the variance in daily precautions was between-person and 15% was within-person. Results from multilevel models with predictors are in Table 5. In the concurrent model, people who scored higher on the knowledge quiz, held higher educational degrees, endorsed less fake news beliefs, and perceived a higher risk of contracting COVID-19 reported more precautions than those who scored lower on the same variables. On a daily basis, increases in the number of in-person interactions and leaving home in the past 24 h were each associated with decreases in precautions. In addition, increases in disruption to daily routine were associated with increases in precautions. Although there was no main effect of information seeking in the concurrent model, there was a significant cross-level interaction between individual differences in perceived risk of contracting COVID-19 and daily fluctuations in information seeking (see Figure 2). Specifically, increases in information seeking were differentially associated with daily precautions depending on one’s perceived risk of developing COVID-19. Those who perceived themselves at low risk appeared to benefit from increases in information and increased their precautions, whereas those who perceived themselves at high risk appeared to reduce their precautions with increased information.

The lagged model (Table 5) includes the previous day’s precautions as a predictor and therefore alters the interpretation of the outcome variable to be the change in precautions from one day to the next. Similar to the concurrent day model, there were significant person-level main effects of the knowledge quiz, educational degree, fake news beliefs, and perceived risk for contracting COVID-19. In addition, the within-person daily effects were also similar. Increases in the number of in-person interactions and leaving home were both associated with decreases in precautions from one day to the next. Unlike the concurrent day model, increases in confidence to avoid contracting COVID-19 were associated with increases in precautions from one day to the next. Finally, the lagged model also revealed a significant cross-level interaction between daily fluctuations in information seeking and individual differences in perceived risk of contracting COVID-19 with a pattern similar to the one found in the concurrent model (Figure 2).

## 4. Discussion

Examining predictors of precautionary behavior early in the pandemic is an important step in understanding decision making related to COVID-19 and may allow us to make predictions about subpopulations to target for current and future information campaigns, such as the most recent push for vaccinations. As predicted by the HBM, information from media and other sources served as cues to action in both studies. That is, we found evidence that low information seeking was indeed related to lower precaution taking behavior, particularly with those who considered themselves low risk for contracting COVID-19. This qualification regarding perceived risk is important because evidence coming out of the CDC and major medical journals suggests that just because a person considers themselves low risk does not mean that (1) they actually are low risk, (2) that they cannot contract COVID-19, and (3) they should not engage in protective behavior [29,30]. Healthy people can both contract and transmit COVID-19 and can be at risk for serious illness and death. Public health campaigns may need to focus on identifying people who do not think they are at risk as a potential target audience for providing fact-based data in multiple media outlets. In addition, when working with people who have a low perceived risk of disease, working to increase quality information gathering may be an outlet for improving behavioral action. Although promoting health behavior change is multifaceted and may require a dynamic approach, these initial steps of increasing access and exposure to valid data and information is important.

Results from the cross-sectional study showed that participants who engaged in more precautionary behavior also had more knowledge about the virus and reported more information seeking. However, there was also a significant interaction between information seeking and perceived risk of developing COVID-19 on endorsed precautions with the relationship between precautions and low information seeking being qualified by level of perceived risk. That is, persons who endorsed low information seeking but perceived themselves as high risk were more likely to also engage in precautionary behavior, while persons who perceived themselves as low risk were less likely to engage in precautionary behavior. It is possible that the people who perceived themselves as being at higher risk did not deem it necessary to continue to seek information because they already were engaged in protective behavior. Increasing cues to action in the form of information-seeking behavior could improve adherence to precautionary behaviors for those who perceive themselves as low risk, which is potentially mediated by increasing perceived risk or worry [31]. Receiving information from different types of sources, including digital media and close family and friends, has been shown to increase COVID-19 risk perception [12,32]. All individuals, however, should be selective when deciding which sources to use to obtain their information and how much time they spend engaging with those sources. Although information seeking was shown to be related to using more precautionary behaviors, the tendency to continuously check the news and pay more attention to negative news (e.g., “doomscrolling”) can lead to discomfort and increased anxiety [33,34], particularly for older men [16]. Given the cross-sectional nature of these data, we used these results as a springboard for examining how information seeking and perceived risk relate to decisions to engage in precautionary behavior on a daily basis.

The novelty of our daily diary approach in Study 2 extends the individual differences identified in Study 1 and in the HBM to the within-person, fluctuating nature of precaution action taking. Depending on level of perceived risk, daily changes in precautions move up and down systematically based on daily changes in information seeking. Those who perceived themselves at low risk appeared to benefit from daily increases in information, whereas those who perceived themselves at high risk appeared to reduce their precautions with daily increased information. Importantly, we were able to establish that these patterns are temporally sequenced. COVID-19 fake news beliefs were also significantly related to perceived ability to avoid COVID-19. If a person did not perceive COVID-19 to be a real threat, their concern about contracting COVID-19 was low. In addition, on days when participants left home, they engaged in fewer precautionary behaviors. The ability to choose to stay home may itself function as a precautionary behavior.

There were some clear consistencies between the two studies with the most important being the interaction between perceived risk and information seeking on precautionary behaviors. In line with the cross-sectional results from Study 1 and the HBM where the likelihood of adopting precautionary health behaviors to prevent contracting and spreading a disease depends on perceived susceptibility of contracting the disease and whether there are effective actions that can be taken to reduce susceptibility or severity if one becomes ill [4], we found that the effect of information was not universal. In Study 2, our results suggest that daily fluctuations in information seeking interacted with individual differences through lagged analyses. That is, increases in information seeking one day were associated with increases in precautions the next day for those who perceived their risk to be low as well as decreases in precautions the next day for those who perceived their risk to be high. Our temporal sequencing suggests that changes in information seeking precede changes in precaution taking. This finding provides strong support in favor of the importance of media messaging in the action of precaution taking. This is further supported, as well as an additional strength of Study 2, in that we were able to show the durability of these within-person effects across a one-month period.

It is important to note that precautions were associated with disruptions to daily routines in both studies. Specifically, people who perceived more disruptions endorsed more precautions in Study 1, and on days when people perceived an increase in the disruptions to their daily routines, they also endorsed more precautions. These findings are in line with the idea that appraisal of stress in the form of disruptions to daily life may influence coping behaviors [35] such as engaging in more precautionary or information-seeking behaviors. Research to date has not addressed the practical implications and the burden of precaution taking. More specifically, research is needed to understand the unique burden of needing to taking precautions when resources are limited, i.e., when there is a global shortage of personal protective equipment. While most people can anecdotally acknowledge the stress that has come with the changes to daily routine due to the increased need for precaution taking, our results confirm that differences between people as well as day-to-day changes in stress appraisals are connected to precautionary behaviors. As psychologists, it is clear that addressing people’s stress and coping, especially as it unfolds on a daily basis surrounding the pandemic, is an important task.

One limitation in both studies was that participants were predominantly white. There are racial/ethnic health disparities related to this pandemic with African Americans and Latinos being disproportionately impacted by COVID-19 [36]. Additional work is needed to understand predictors of precautionary behaviors among communities experiencing the most severe health risks and outcomes. In addition, the majority of participants in the daily diary study (Study 2) were women, which may limit generalization. Political ideology emerged as a potential factor of precautionary actions as the COVID-19 pandemic progressed. Political ideology was not as strong of a factor at the time the study was initiated and therefore was not included. Finally, the HBM is a cognitive model that does not take into account emotional aspects of behavior [37]. Future research should also consider emotional constructs, such as fear, that may influence both beliefs and behaviors. In addition, we used the HBM as a foundation to interrogate individual-level precautionary behaviors, but it would be useful for future work to incorporate community-level factors as well. The COM-B model for behavior change which uses three constructs (i.e., capability, opportunity, and motivation) as potential drivers of change may provide a useful framework for future studies and interventions focused on enhancing both precautionary and protective behaviors [38]. For instance, the burden of taking precautions when there is a stark unavailability of the resources needed to be proactive speaks more to opportunity than to information seeking [39]. We intend to incorporate these ideas into our forthcoming follow-up study with the participants from Study 2 to see how the patterns observed in the current study may remain stable or change over time.

## 5. Conclusions

Based on these results, we suggest that public health measures should be focused on helping people accurately appraise their own risk profile and to encourage information seeking from valid sources as a way of increasing compliance with recommended precautionary behaviors and bolster coping strategies. In addition, understanding how people develop their risk perceptions under stressful conditions such as a pandemic, particularly in the face of low information seeking, is a potentially fruitful area of exploration for future studies. Changing people’s beliefs about conspiracies and fake news is an important challenge for psychologists and public health officials.

While there is considerable literature and debate about how to best address misinformation and misrepresented scientific facts in the media, the pathway to addressing this remains unclear, particularly given the freedoms of press speech afforded individuals as well as companies in the United States. Macro-level suggestions for addressing this include increasing consumer protection regulations and encouraging the use of local news, which tends to be more accountable to communities [40]. Micro-level suggestions, based on the two studies reported here as well as other pandemic-related publications, include improving risk profile appraisals and increasing scientific information seeking [41].

## Figures and Tables

**Table 1:** Sample Characteristics.

	Study 1			Study 2		
Variables	Mean (SD)	Range	Valid Percent	Mean (SD)	Range	Valid Percent
Age	38.74 (11.51)	20–79		64.29 (5.20)	55–79	
Gender						
Male			59.9			32.2
Female			40.1			67.8
Race						
American Indian			0.3			0.4
Asian			5.4			1.5
Black or African American			8.0			2.3
White			83.7			92.7
Other			0.3			1.1
More than one race			2.1			1.9
I do not wish to answer			0.3			
Income						
$10,000 or less			2.8			2.7
$10,000 to $25,000			11.3			16.5
$25,000 to $50,000			34.0			35.2
$50,000 to $100,000			39.3			33.3
$100,000 or more			12.6			12.3

Note. Income was measured on a scale ranging from 1 ($10,000 or less per year) to 5 ($100,000 or more per year). The US population in 2020 was 61.6% White, 12.4% Black, 6% Asian, 18.7% Hispanic, 1.1% American Indian or Alaska Native, 6.2% some other race, and 10.2% more than one race. These statistics were taken from the 2020 United States Census. The total of all race categories is based on the number of responses; therefore, it adds to more than 100%.

**Table 2:** Percentages of Endorsed Precautions.

	Percent	
Precaution	Study 1	Study 2
Avoid people who cough and/or sneeze	95	96
Covering mouth and nose when coughing and sneezing	95	97
Avoid large gatherings of people	96	95
Avoid small gathers of people except family	92	88
Wash your hands more often	94	92
Use hand sanitizer more often	83	67
Avoid people who are in contact with infected people	95	95
Avoid public transportation	93	96
Avoid school and/or work	79	76
Avoid public spaces	83	75
Avoid travel to infected areas	93	95
Avoid any travel	85	83
Use disinfectant on surfaces	88	79
Wear a mask	60	54

**Table 3:** Descriptive Statistics of Study Variables.

	Study 1				Study 2			
Variables	Mean	SD	Min	Max	Mean	SD	Min	Max
Precautions	87.89	16.57	8.33	100	84.84	18.14	0	100
COVID-19 Knowledge	18.57	4.51	3	23	19.85	2.80	5	23
Degree	6.64	1.55	2	10	6.56	1.86	2	10
Age	38.74	11.52	20	79	64.61	5.13	55	79
Income	3.48	0.95	1	5	3.37	0.94	1	5
Information Seeking	2.38	0.92	1	5	1.37	0.32	1	3.8
In-Person Interactions	3.79	29.43	0	800	4.62	18.30	0	500
COVID-19 Fake News Beliefs	1.13	1.58	0	6	0.86	1.35	0	6
Ability to Avoid COVID-19	3.62	1.02	1	5	3.83	0.88	1	5
Risk of Developing COVID-19	2.91	1.25	1	5	2.07	0.94	1	4
Disruption to Daily Routine	2.72	0.98	1	4	2.69	1.03	1	5
Left Home	--	--	--	--	0.48	0.50	0	1

Note. Precautions are calculated as a percentage of total precautions endorsed. COVID-19 Knowledge Quiz is calculated as total correct out of 23. Highest educational degree was measured on a scale ranging from 1 (Grades 1–8) to 10 (Ph.D., M.D. or other professional degree). Income was measured on a scale ranging from 1 ($10,000 or less per year) to 5 ($100,000 or more per year). Left home that day was only measured in Study 2.

**Table 4:** Correlations Among Study Variables.

Variables	1	2	3	4	5	6	7	8	9	10	11	12
1. Precautions	--	.16 ***	.07	−.03	.10 **	.09 *	−.10 **	−.13 ***	−.02	.08 *	.19 ***	--
2. COVID-19 Knowledge	.31 ***	--	−.17**	.10 **	.03	−.59 ***	−.08 *	−.53 ***	−.16 ***	−.04	−.03	--
3. Degree	−.08	.04	--	−.03	.25 ***	.28 ***	.01	.19 ***	.05	.06	.06 ***	--
4. Age	−.02	−.03	.09	--	−.03	−.07	−.02	.01	.04	.01	−.05	--
5. Income	.05	.00	.16 *	−.07	--	.02	−.05	−.08 *	.01	.04	.01 **	--
6. Information Seeking	−.01	−.29 ***	−.03	−.10	.14 *	--	.08 *	.40 ***	.19 ***	.16 ***	.22 ***	--
7. In-Person Interactions	−.14 *	.02	.04	−.02	−.05	−.06	--	−.01	.00	−.05	−.02	--
8. COVID-19 Fake News Beliefs	−.47 ***	−.43 ***	−.04	.04	.00	.10	.10	--	.25 ***	−.08 *	−.05	--
9. Ability to Avoid COVID-19	−.12	−.11	−.08	.08	.15 *	−.04	−.04	−.17 **	--	−.32 ***	−.07	--
10. Risk of Developing COVID-19	.25 **	.08	.02	−.06	−.06	.08	.08	.07	−.31 ***	--	.26 ***	--
11. Disruption to Daily Routine	.11	.09	.12	−.08	.17 **	.22 **	.22 **	−.08	−.16 *	−.30 ***	--	--
12. Left Home	−.19 *	.02	.05	.07	.17 *	−.20 **	−.20 **	.17 *	.11	.10	−.13 *	--

Note. Study 1 above the diagonal. Study 2 below the diagonal. Variable “Left Home” was not administered in Study 1. * *p* < .05. ** *p* < .01. *** *p* < .001.

**Table 5:** Final Models for Predicting Endorsed Precautions.

Fixed Effects	Study 1 B (SE B)	Study 2—Concurrent B (SE B)	Study 2—Lagged B (SE B)
Intercept	88.40 (0.60) ***	58.10 (17.25) **	45.43 (13.58) **
Previous day’s Precautions			23.19 (1.36) ***
COVID-19 Knowledge	0.82 (0.17) ***	1.18 (0.43) **	0.86 (0.34) *
Degree	0.19 (0.40)	−1.12 (0.53) *	−0.92 (0.42) *
Age	−0.03 (0.05)	0.10 (0.19)	0.07 (0.15)
Income	0.85 (0.62)	1.10 (1.02)	0.71 (0.81)
Individual Information Seeking	4.16 (0.84) ***	−0.16 (0.68)	−1.02 (0.70)
Daily Information Seeking		2.22 (2.59)	2.76 (2.26)
In-Person Interactions	−0.05 (0.02) **	−0.05 (0.01) ***	−0.04 (0.01) ***
COVID-19 Fake News Beliefs	−0.64 (0.45)	−4.93 (0.84) ***	−3.85 (0.66) ***
Ability to Avoid COVID-19	0.50 (0.63)	0.45 (0.32)	0.81 (0.33) *
Risk of Developing COVID-19	0.57 (0.54)	2.95 (1.42) *	2.55 (1.25) *
Disruption to Daily Routine	1.92 (0.62) **	0.41 (0.20) *	0.39 (0.21)
Left Home		−3.07 (0.26) ***	−3.06 (0.26) ***
Information Seeking*Perceived Risk	−1.25 (0.46) **	−1.46 (0.66) *	−1.36 (0.66) *

Note. The concurrent day model explained 4% of the within-person and 29% of the between-person variance in daily precautions. The lagged model explained 11% of the within-person and 58% of the between-person variance in daily precautions. * *p* < .05. ** *p* < .01. *** *p* < .001.

## Data Availability

The studies and the statistical analysis plans were not pre-registered. The data are not publicly available online but will be made available upon reasonable request.
